# Current status of mesenchymal stem cell therapy for immune/inflammatory lung disorders: Gleaning insights for possible use in COVID‐19

**DOI:** 10.1002/sctm.20-0186

**Published:** 2020-06-11

**Authors:** B. Linju Yen, Men‐Luh Yen, Li‐Tzu Wang, Ko‐Jiunn Liu, Huey‐Kang Sytwu

**Affiliations:** ^1^ Regenerative Medicine Research Group Institute of Cellular & System Medicine, National Health Research Institutes (NHRI) Zhunan Taiwan; ^2^ Department of Obstetrics/Gynecology National Taiwan University (NTU) Hospital & College of Medicine, NTU Taipei Taiwan; ^3^ National Institute of Cancer Research, NHRI Tainan Taiwan; ^4^ National Institute of Infectious Diseases & Vaccinology, NHRI Zhunan Taiwan; ^5^ Department & Graduate Institute of Microbiology and Immunology National Defense Medical Center Taipei Taiwan

**Keywords:** ARDS, asthma, bacterial pneumonia, clinical trial, COPD, COVID‐19, cytokine storm, idiopathic pulmonary fibrosis, influenza, mesenchymal stem cells

## Abstract

The broad immunomodulatory properties of human mesenchymal stem cells (MSCs) have allowed for wide application in regenerative medicine as well as immune/inflammatory diseases, including unmatched allogeneic use. The novel coronavirus disease COVID‐19 has unleashed a pandemic in record time accompanied by an alarming mortality rate mainly due to pulmonary injury and acute respiratory distress syndrome. Because there are no effective preventive or curative therapies currently, MSC therapy (MSCT) has emerged as a possible candidate despite the lack of preclinical data of MSCs for COVID‐19. Interestingly, MSCT preclinical data specifically on immune/inflammatory disorders of the lungs were among the earliest to be reported in 2003, with the first clinical use of MSCT for graft‐vs‐host disease reported in 2004. Since these first reports, preclinical data showing beneficial effects of MSC immunomodulation have accumulated substantially, and as a consequence, over a third of MSCT clinical trials now target immune/inflammatory diseases. There is much preclinical evidence for MSCT in noninfectious—including chronic obstructive pulmonary disease, asthma, and idiopathic pulmonary fibrosis—as well as infectious bacterial immune/inflammatory lung disorders, with data generally demonstrating therapeutic effects; however, for infectious viral pulmonary conditions, the preclinical evidence is more scarce with some inconsistent outcomes. In this article, we review the mechanistic evidence for clinical use of MSCs in pulmonary immune/inflammatory disorders, and survey the ongoing clinical trials—including for COVID‐19—of MSCT for these diseases, with some perspectives and comment on MSCT for COVID‐19.

## Graphical abstract

Mesenchymal stem cell (MSC) therapy for immune/inflammatory pulmonary disorders. Broad immunomodulatory properties of human MSCs and MSC‐derived products allow for therapeutic use in noninfectious—including chronic obstructive pulmonary disease, asthma, and idiopathic pulmonary fibrosis—and a number of infectious immune/inflammatory lung disorders.

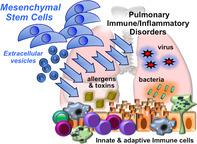

**Significance statement**

Human mesenchymal stem cell (MSC) immunomodulation is clinically relevant, allowing for allogeneic use and broad application in immune/inflammatory diseases including severe pulmonary injury and inflammation such as acute respiratory distress syndrome (ARDS) associated with bacterial and viral infections. Recently, MSC therapy (MSCT) is seen as a possible candidate for treating severe ARDS and cytokine storm from the novel coronavirus COVID‐19, despite a lack of preclinical data. This study reviews the mechanistic evidence for MSCT in pulmonary immune/inflammatory disorders, and surveys the ongoing clinical trials including for COVID‐19, with specific commentary on MSCT for COVID‐19.

## 1 INTRODUCTION

Human mesenchymal stem/stromal cells (MSCs) are multilineage somatic progenitors with broad immunomodulatory properties. Since initial isolation from the bone marrow (BM), MSCs have been found in numerous adult and fetal‐derived organs/tissues such as adipose tissue, dental pulp, umbilical cord, and placenta.^1^ In addition to trilineage paraxial mesodermal differentiation capacity toward bone, cartilage, and fat, the immunomodulatory properties of MSCs not only allow for expansion of therapeutic use from regenerative medicine to immune‐ and inflammation‐related diseases, but also for third party allogeneic use.^2^

The first published full report on clinical use of MSCs for immune/inflammatory disease was in 2004, in which allogeneic haploidentical bone marrow mesenchymal stem cell (BMMSC) infusions were given for a pediatric patient with acute refractory graft‐vs‐host‐disease (GVHD).^3^ Of note, median survival at that institution was a mere 2 months for the other 24 patients with similarly severe GVHD, while this patient remained well 1 year after MSC treatment. Surprisingly, prior to this clinical case report, there were only a handful of studies demonstrating MSC immunomodulation, with only one study showing in vivo data of prolonged skin engraftment.^4^, ^5^, ^6^ Since then, MSC immunomodulation has shown to be broad‐based, best detailed for CD4 lymphocytes but also for dendritic cells and natural killer cells.^7^, ^8^ The immunomodulatory properties are clinically relevant, as evidenced by the increasing proportions of MSC trials focusing on immune/inflammatory diseases which in recent years has accounted for approximately one‐third of the trials.^9^

**FIGURE 1:**
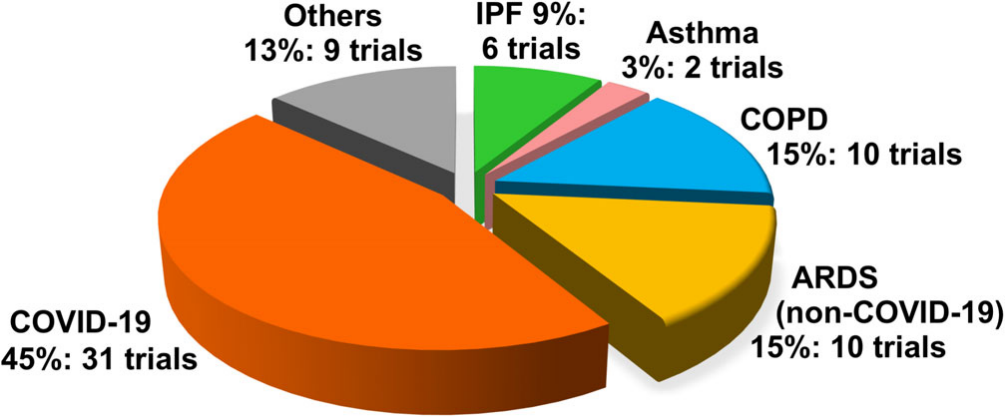
Current disease distribution of clinical trials using human MSC for immune/inflammatory pulmonary disorders. Numbers of MSC clinical trials for various immune/inflammatory pulmonary disorders as registered on the NIH Clinical Trial Registry website (https://ClinicalTrials.gov/) as accessed on May 2020. ARDS, acute respiratory distress syndrome; COPD, chronic obstructive pulmonary disease; COVID‐19, coronavirus disease 2019; IPF, idiopathic pulmonary fibrosis; MSC, mesenchymal stem cell

One of the earliest reports demonstrating MSC immunomodulation was in reduction of bleomycin‐induced pulmonary inflammation in mice.^10^ It comes as somewhat of a surprise that clinical use of MSCs for lung diseases has been relatively slow to start, with most trials initiated in 2015. Moreover, it had been known for over a decade that intravenous delivery of MSCs—the most typical method of intervention for any cell therapy—results in the overwhelming majority of cells (80%~90%) lodging in the lungs which is further increased with inflammation.^10^, ^11^ Hence, there is discussion that MSC therapy (MSCT) may be particularly useful in immune/inflammatory pulmonary conditions.^12^ However, clinical trials for these diseases were still relatively few until this year: as of 17 May 2020, out of 68 MSC trials for lung immune/inflammatory diseases, 31 trials are specifically for COVID‐19 as registered on the NIH Clinical Trial Database (https://ClinicalTrials.gov/) (Figure 1). Due to the rapid global spread of COVID‐19, the high mortality rate of those with severe disease, and no proven effective therapies as of yet, a desperate search for possible treatments is ongoing.^13^ MSCT is clearly one such attempt, with new trials being added almost daily despite the lack of COVID‐19‐related preclinical data. In this review, we will examine the mechanistic evidence for clinical use of MSCs in pulmonary immune/inflammatory disorders, and survey the ongoing clinical trials—including for COVID‐19—of MSCT for these diseases, with some perspectives and comments on MSCT for COVID‐19.

## 2 PRECLINICAL DATA OF MSCT FOR NONINFECTIOUS PULMONARY IMMUNE/INFLAMMATORY DISORDERS

**FIGURE 2:**
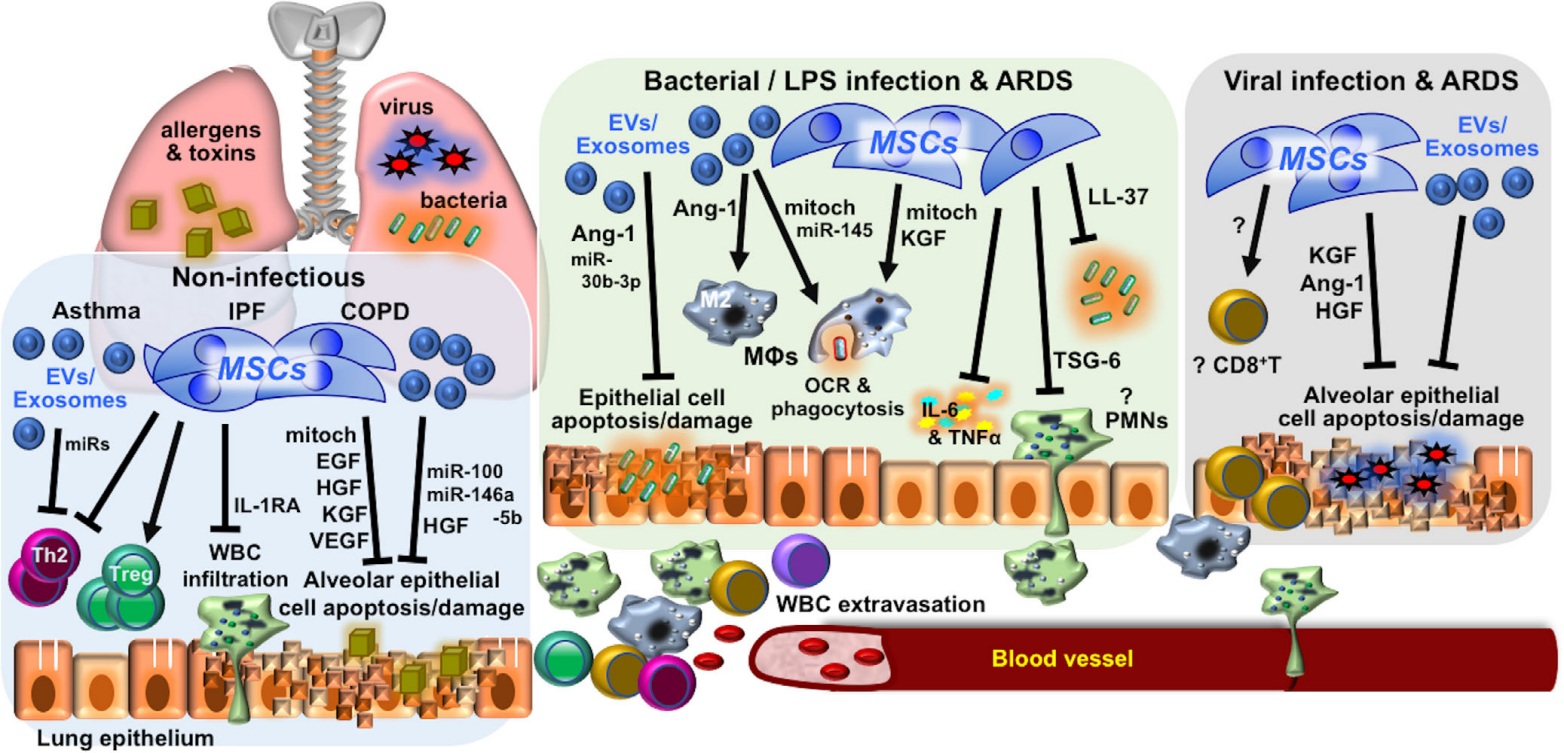
Mechanisms involved in MSC therapy for immune/inflammatory pulmonary disorders. Mechanisms reported in in vivo preclinical studies of MSC therapy for immune/inflammatory lung diseases of non‐infectious etiology—including asthma, IPF, and COPDs—and infectious etiology—including bacterial and/or LPS and viral infection and related ARDS. Detailed descriptions can be found in the text. Ang‐1, angiopoietin‐1; ARDS, acute respiratory distress syndrome; COPD, chronic obstructive lung disease; EGF, epidermal growth factor; EV, extracellular vesicles; HGF, hepatocyte growth factor; IL‐1RA, interleukin‐1 receptor antagonist; IPF, idiopathic pulmonary fibrosis; KGF, keratinocyte growth factor; LPS, lipopolysaccharide; MΦ, macrophage; miRs, microRNAs; mitoch, mitochondria; MSC, mesenchymal stem cell; OCR, oxygen consumption rate; PMNs, polymorphonuclear leukocytes/neutrophils; Th2, T helper type 2 lymphocytes; TNF‐α, tumor necrosis factor‐α; Treg, regulatory T lymphocytes; TSF‐6, TNF‐stimulated gene 6 protein; VEGF, vascular endothelial growth factor; WBCs, white blood cells

The lungs are in direct exposure to the external environment, requiring constant immune surveillance by the native epithelial cells and resident alveolar macrophages for homeostasis and health.^14^ Immune dysregulation and inflammation, therefore, are common components in both infectious and many noninfectious pulmonary diseases, such as obstructive diseases including chronic obstructive pulmonary diseases (COPDs), in which injury is mainly mediated by cytotoxic T cells and neutrophils, and asthma, where type 2 helper T (Th2) lymphocytes and eosinophils are more predominant.^15^ In restrictive diseases such as idiopathic pulmonary fibrosis (IPF), resident alveolar macrophages appear critical in mediating the fibrosis.^16^ Despite differences in underlying processes for these diseases, MSCs have generally been shown to have beneficial effects in preclinical studies. Numerous rodent studies on COPD demonstrated that MSC infusion decrease inflammation and parenchymal damage^17^ with a number of reports showing MSC paracrine factors including epidermal growth factor, hepatocyte growth factor (HGF),^18^, ^19^, ^20^, ^21^ vascular endothelial growth factor,^17^, ^22^, ^23^ and keratinocyte growth factor (KGF)^24^ to be involved (Figure 2, left box). Rodent studies of asthma demonstrate that MSC induction of CD4 regulatory T cells (Tregs), which are immunomodulatory CD4 cells, is critical in decreasing Th2 responses^25^, ^26^ and Th2 cytokines interleukin‐4 (IL‐4), IL‐5, and IL‐13 as well as immunoglobulin E levels to ameliorate disease severity^27^, ^28^; one recent study implicated MSC transfer of mitochondria in this process.^29^ Surprisingly, in these studies on asthma, no specific MSC paracrine factor was identified, but more recent reports implicate that MSC‐expressed microRNA and exosomes can improve disease outcome.^30^, ^31^

Despite transforming growth factor‐β being a prominent paracrine factor of MSCs^7^, ^32^ and also known for strongly inducing fibrosis, MSCT appears to be efficacious even for fibrotic pulmonary conditions, as evidenced by the early study of MSC efficacy for bleomycin‐induced lung fibrosis, a preclinical disease model of IPF.^10^ MSC‐secreted IL‐1 receptor antagonist (IL‐1RA) was subsequently shown by the same group to be the paracrine factor involved.^33^ Moreover, MSCs may enhance resident lung bronchioalveolar stem cells to repair and regenerate healthy lung parenchyma.^34^ There has also been a growing number of reports using MSCs other than BMMSCs including umbilical cord MSCs (UCMSCs),^35^, ^36^ adipose‐derived MSCs (AdMSCs),^37^, ^38^ and placental MSCs.^39^ More recent studies also implicate MSC‐secreted factors of HGF and exosomes in mediating the antifibrotic effects.^40^, ^41^, ^42^

## 3 PRECLINICAL DATA ON MSCT FOR BACTERIAL PNEUMONIA AND COMPLICATIONS

The immunomodulatory effects of MSCs may lead one reasonably to avoid using these cells in infectious diseases, especially bacterial infectious since a strong effector response is required for clearance of these rapidly growing microorganisms. But surprisingly, the preclinical data have been rather consistent on MSCs actually enhancing antibacterial processes and decreasing overexuberant immune responses leading to pulmonary injury and acute respiratory distress syndrome (ARDS), a complication which is still associated with high morbidity and mortality.^43^ In rodent models of lung injury using either lipopolysaccharide, a component of Gram‐negative bacterial cell wall, or pneumonia induced by live bacteria (mainly *Escherichia coli*), numerous studies have shown that MSCs, MSC‐conditioned medium, or MSC‐exosomes suppress inflammatory cell infiltration, decrease pro‐inflammatory cytokine levels including tumor necrosis factor‐α (TNF‐α) and IL‐6, reverse pulmonary tissue damage, and improve survival through numerous paracrine factors including TNF‐stimulated gene 6 protein (TSG‐6),^44^ angiopoietin‐1,^45^, ^46^ LL‐37,^47^ lipocalin‐2,^48^ KGF,^46^, ^48^, ^49^ and microRNAs (Figure 2, middle box).^50^ Beneficial effects of MSCT in ex vivo human lung injury/bacterial infection models were seen as well.^51^ Similar to a report for asthma, mitochondrial transfer from MSCT—either directly or through exosomes—decreased pulmonary injury and improved macrophage energetics and antibacterial functions.^52^, ^53^ Other studies have also found that MSCs modulate macrophages from an M1 inflammatory phenotype to a more immunomodulatory M2 phenotype,^54^, ^55^, ^56^ as has been shown in nonpulmonary in vivo models.^57^, ^58^ It is surprising, however, that no in‐depth investigation of MSCs with neutrophils, the first‐line and critical leukocyte involved in bacterial clearance, was carried out any of these animal studies, since in vitro reports and one in vivo sepsis model have shown that MSCs preserve neutrophil viability and antibacterial functions.^59^, ^60^, ^61^ But overall, these preclinical studies of bacterial‐related lung injury/pneumonia consistently demonstrate that MSCT improves bacterial clearance and pulmonary tissue repair to impact survival.

## 4 PRECLINICAL DATA OF MSCT FOR VIRAL PNEUMONIA AND COMPLICATIONS

Overall, reports on MSCT for viral infections are relatively scarce. Most of the in vitro studies have been on the H1N1 influenza virus using MSCs from many different organisms, finding that MSCs can be infected with resultant cell lysis and death.^62^, ^63^ For in vivo studies, there are currently only six reports which have examined intravenous MSCT for viral pneumonitis/pneumonia, all focusing on influenza. In the first two studies on the subject, the outcome was negative. Both studies evaluated syngeneic murine as well as allogeneic human BMMSC treatment in mice infected with pulmonary mouse‐adapted H1N1 and/or swine H1N1, with no improvement in pulmonary inflammation or survival seen.^64^, ^65^ In the four other more recent reports, however, pulmonary inflammation was improved overall, with survival seen to improve in two out of the three studies which evaluated this endpoint; no specific factor was shown to be responsible (Figure 2, right box). Interestingly, all three reports which evaluated survival used non‐H1N1 subtypes. The only one beneficial report using H1N1 was a porcine study in which in vivo infection with swine H1N1 in 8‐week‐old pigs improved lung inflammation after intratracheal administration of syngeneic BMMSC extracellular vesicles; survival was not evaluated.^66^ A report using H9N2 found syngeneic BMMSC treatment suppressed infection and improved survival in infected mice,^67^ whereas another study using H5N1 found that conditioned medium and exosomes from human UCMSCs but not BMMSCs improved lung injury in infected mice partly due to two paracrine factors, angiopoietin‐1 and HGF, but survival was only minimally improved.^68^ A more elaborate murine study found that human BMMSCs reduce H5N1‐induced lung injury and survival but only in aged mice, partially through the paracrine factors of angiopoietin‐1 and KGF; the improved response in aged mice (8‐12 months old) but not young mice (6‐8 weeks old) to MSCT was in part attributed to more severe disease in these aged hosts, which may allow for exogenous MSCT to exert a more obvious benefit.^69^ This report also further discussed that the tissue reparative properties of MSCs may only be apparent with the severe damage caused by highly pathogenic influenza subtypes including H5N1, which is not seen with the less pathogenic H1N1 subtype. The collective results of these in vivo studies, while few, would seem to support this viewpoint. Such differences in the infecting viral subtype and host conditions are unfortunately rarely tackled in preclinical studies, but in clinical practice, differences in patient profiles, including age and sex, are known to highly influence disease progression and outcome—as is strikingly evident with COVID‐19, with higher positivity rates and worse outcome in men, and significantly higher mortality in the elderly and those with underlying chronic diseases.^70^, ^71^ Preclinical studies clearly should pay attention to such parameters for improved clinical use and outcome.

One important point to keep in mind in interpreting these few in vivo MSCT‐virus reports is the existing data on MSC interactions with CD8 cells, which have a more critical role in viral infections.^72^ Surprisingly, very few reports have studied MSC‐CD8 interactions, in contrast to the several hundred reports on MSC‐CD4 interactions. While some reports show inconsistent MSC regulation of effector CD8 cell types),^73^, ^74^ most studies including data from our lab found that MSCs suppress CD8 T proliferation and cytotoxicity.^75^, ^76^, ^77^ Interestingly, one in vitro study found MSCs to inhibit proliferation of virus‐specific CD8 cells, leading the authors to comment that the use of MSCs may therefore compromise viral T‐cell immunity.^78^ However, in reports of clinical MSCT in GVHD patients, MSCT generally did not suppress viral‐specific T‐cell responses in patients, despite demonstrating strong in vitro immunomodulatory effects across CD4 and CD8 lymphocytes.^79^, ^80^ While existing in vitro and in vivo patient‐derived reports demonstrate discrepant information, the clinical studies offer some reassurance on the safety of MSCT but also reveal the gap in understanding the efficacious mechanisms—if any—of MSCT in patients. Clearly, preclinical in vivo studies with focus on elucidating specific populations of leukocyte‐MSC interactions during all steps of viral pneumonia/lung injury are urgently needed to provide better insight for clinical use.

## 5 CURRENT CLINICAL TRIALS OF MSCT FOR PULMONARY IMMUNE/INFLAMMATORY DISEASES

To date, 68 clinical studies using MSCs for pulmonary immune/inflammatory disorders have been registered (Figure 1 and Table S1 for detailed information on each trial). The most commonly targeted disease is COVID‐19 with 31 trials. MSCT for ARDS other than for COVID‐19 and COPD are the next two most commonly targeted diseases, with 10 trials each. There are six trials for IPF, and two trials for asthma; the rest of the nine trials include trials for cystic fibrosis, lung transplantation, pneumoconiosis, radiation‐caused injury, and unspecified lung injury.

**FIGURE 3:**
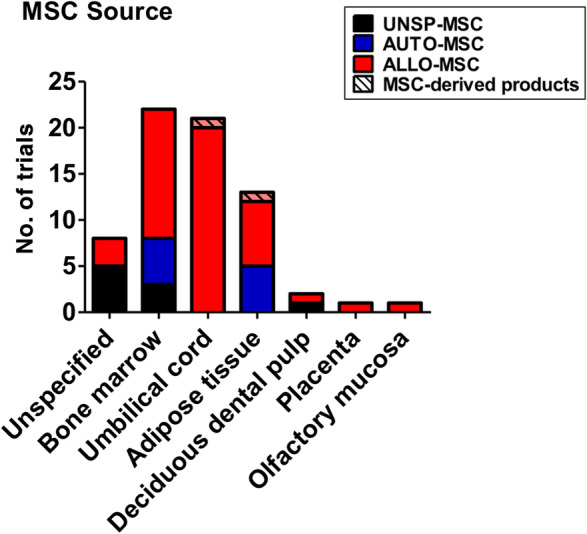
Sources of human MSCs used in immune/inflammatory lung disease clinical trials. Number of trials using different sources of human MSCs is shown, and whether sources are autologous (AUTO‐MSC), allogeneic (ALLO‐MSC), unspecified (UNSP‐MSC), and/or noncell exosomes/conditioned medium (MSC‐derived products). Data accessed on May 2020 from the NIH Clinical Trial website (https://ClinicalTrials.gov/). MSC, mesenchymal stem cell

**FIGURE 4:**
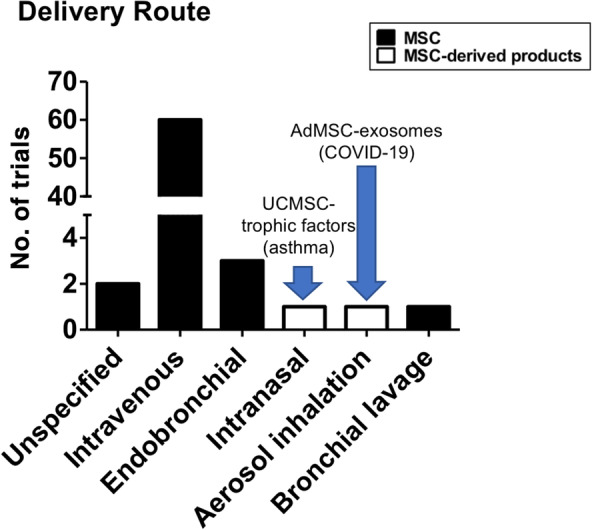
Immune/inflammatory lung disease clinical trials. Number of trials using various methods of MSC administration. Data accessed on May 2020 from the NIH Clinical Trial website (https://ClinicalTrials.gov/). MSC, mesenchymal stem cell

MSC sources used are broad (Table 1 and Figure 3), with the two most common types being BMMSCs (22 trials) and UCMSCs (20 trials), then AdMSCs with 12 trials. A few trials use either dental pulp MSCs (two trials), placenta‐MSCs (one trial), and olfactory mucosa MSCs (one trial); eight trials did not specify MSC source. As testament to the strong evidence for MSC immunomodulation, the majority of trials use allogeneic MSCs (49 trials, includes two trials using conditioned medium and exosomes), with all UCMSC trials being allogeneic; 10 trials use autologous sources which are either BMMSCs or AdMSCs, and nine trials were unspecified. There are only two trials using MSC‐derived products such as conditioned medium or exosomes rather than the cells themselves, and these products are derived from allogeneic AdMSCs or UCMSCs. Trials tend to be at early phases, with 30 being phase 1 trials, 17 being combined phase 1/2 trials, 14 trials in phase 2, 2 trials being a combined phase 2/3 trials, and 1 trial in phase 3; 3 trials are unspecified. As expected, the overwhelming majority of trials deliver MSCs intravenously (60 trials) but surprisingly, except for two trials which did not specified delivery method, the remaining six trials deliver MSCs to the lungs more directly, either through intratracheal/endobronchial delivery (three trials), intranasal delivery (one trial using UCMSC‐conditioned medium), aerosolized inhalation (one trial using AdMSC‐derived exosomes), or bronchial lavage (one trial) (Figure 4).

**TABLE 1:** Cell source and trial phase of MSC clinical trials for immune/inflammatory lung diseases

MSC source	Total %	Total no.	No. of clinical trial phases					
			?	1	1&2	2	2&3	3
Unspecified	11.8	8	0	5	0	2	1	0
Bone marrow	32.4	22	0	11	2	6	2	1
Umbilical cord	29.4	20	3	7	8	2	0	0
Adipose tissue	17.6	12	0	3	5	4	0	0
Deciduous dental pulp	2.9	2	0	1	1	0	0	0
Placenta	1.5	1	0	1	0	0	0	0
Olfactory mucosa	1.5	1	0	0	1	0	0	0
MSC‐derived products^a^	2.9	2	0	2	0	0	0	0
Total no. of clinical trial phases		68	3	30	17	14	3	1
Total % of clinical trial phases			4.4	44.1	25.0	20.6	4.4	1.5

^a^ Exosomes or trophic factors collected from conditioned medium.

Abbreviation: MSC, mesenchymal stem cell.

As most of these trials are just starting or still ongoing, there are very few published results available. An important publication was of the earliest MSCT trial for lung diseases which was started in 2008: a large phase 2 multicenter trial of 62 patients evaluating allogenic BMMSCs for COPD, where safety was demonstrated but efficacy was less clear.^81^ Several phase 1 trials using various tissue‐source allogeneic MSC infusions have published results: two trials on ARDS, one using AdMSCs^82^ and one using BMMSCs^83^; and two trials on IPF, one using placental‐derived MSCs^84^ and one using BMMSCs.^85^ All four reports demonstrated safety, but efficacy was weak at best. The strong evidence shown in preclinical animal studies has not yet been seen in these published human trials, and this may be a consequence of the very small patient numbers (approximately 8‐12 patients) in the majority of the trials, and also because many of these are just phase 1 trials, which are focused on safety rather than efficacy. Interestingly, a case report of allogeneic third party BMMSC treatment (multiple infusions) in two patients with severe ARDS from different causes demonstrated very good outcome and recovery for both patients, with inflammatory markers IL‐6 and interferon‐γ decreasing the day after initial MSC infusion in less critically ill patient.^86^ One setback occurred in this patient who had H1N1‐related ARDS, with development of bacterial pneumonia 5 days after MSC administration which responded to antibiotics treatment. While causation cannot be concluded from just this one case, the risk of infection in MSCT with its strong immunomodulation is nevertheless a possibility, as the authors also cautioned. Close follow‐up of patients undergoing MSC treatment especially in the critically ill—such as patients with ARDS—is clearly warranted.

Very recently, an open‐label trial from China on MSCT for H7N9‐induced ARDS was published in February 2020,^87^ in which 17 critically ill patients were given multiple infusions of allogeneic (single‐donor) menstrual blood‐derived MSCs, with 44 patients in the control, nontreated group. Within these two groups, 14 patients (82%) in the MSC‐group were mechanically ventilated, compared to 31 patients (66%) in the control group. No adverse reaction to MSCT was reported, and results showed a lower mortality in the MSCT group with three deaths (82% survival) compared to 24 deaths (46% survival) in the control group. No immunological analyses were performed, but clinical lab data showed no difference in total white blood cell count, neutrophil count, or lymphocyte count at discharge between MSCT and control groups; however, procalcitonin, an inflammatory index marker, was significantly decreased in MSCT patients compared to control patients at discharge. While this data is encouraging, more detailed information on patient parameters, as well as the rationale for multiple infusions—not similarly done across MSCT patients—and immunological status at the time of these of the infusions should have been evaluated for a better understanding of MSC effects during ARDS and cytokine storm. Moreover, there is no information regarding the MSCs—which are from a less commonly used source—used in this trial: no references were cited and no characterization was performed. There is growing preclinical data suggesting that “not all MSCs are equal,” with different tissue sources of MSCs expressing different factors at varying levels as well as having functional differences, with a recent review finding BM and UCMSCs more effective than AdMSCs at reducing mortality in preclinical acute lung injury models.^88^ There has also been much discussion on whether the use of fresh vs cryopreserved MSCs would have therapeutic implications.^89^ In the six published clinical reports which all used allogeneic sources, two studies used freshly cultured MSCs^84^, ^85^ whereas the other four studies used previously cryopreserved MSCs^81^, ^82^, ^83^; no clear difference in efficacy could be easily discerned. In addition, the delivery method and dose of cells given, and whether multiple doses should be given, as well as cell numbers used are also critical parameters that likely impact efficacy, but all are difficult to test in human studies. Further accumulation of preclinical data investigating these parameters is urgently needed for better tailoring of specific tissue‐source MSCs, MSC preparation, as well as dosing regimens in clinical use to improve outcome.

## 6 MSCT TRIALS FOR COVID‐19: THE DEVIL MAY BE IN THE DETAILS

One startling discovery is that the majority of MSC clinical trials for immune/inflammatory lung disorders currently are for COVID‐19, a disease that did not exist 6 months ago.^90^ As of this writing, there are over 4.5 million people diagnosed with COVID‐19 and over 310 000 deaths. Novel coronaviruses like SARS‐CoV‐2, the cause of COVID‐19, have cropped up previously in the past three decades—SARS‐CoV in 2003, and MERS‐CoV in 2012—but these two previous outbreaks did not result in global pandemics despite having much higher mortality rates than COVID‐19.^91^ This appears to be due to both the higher infectivity of COVID‐19 as well as infectivity during the asymptomatic phase, resulting in a worldwide crisis which is still escalating.^92^ While most infected patients have mild disease with symptoms mainly of fever and cough, those with severe disease progress to ARDS with concomitant cytokine storm requiring mechanical intubation, with many patients still unable to survive despite such aggressive intervention.^70^, ^71^ The sheer numbers of infected patients with severe disease has overwhelmed healthcare systems around the world, leading to a frenzied search for effective treatments which currently is still largely lacking.^13^ As a result, there are an unprecedented number of clinical trials for COVID‐19, including trials using MSCs.

**FIGURE 5:**
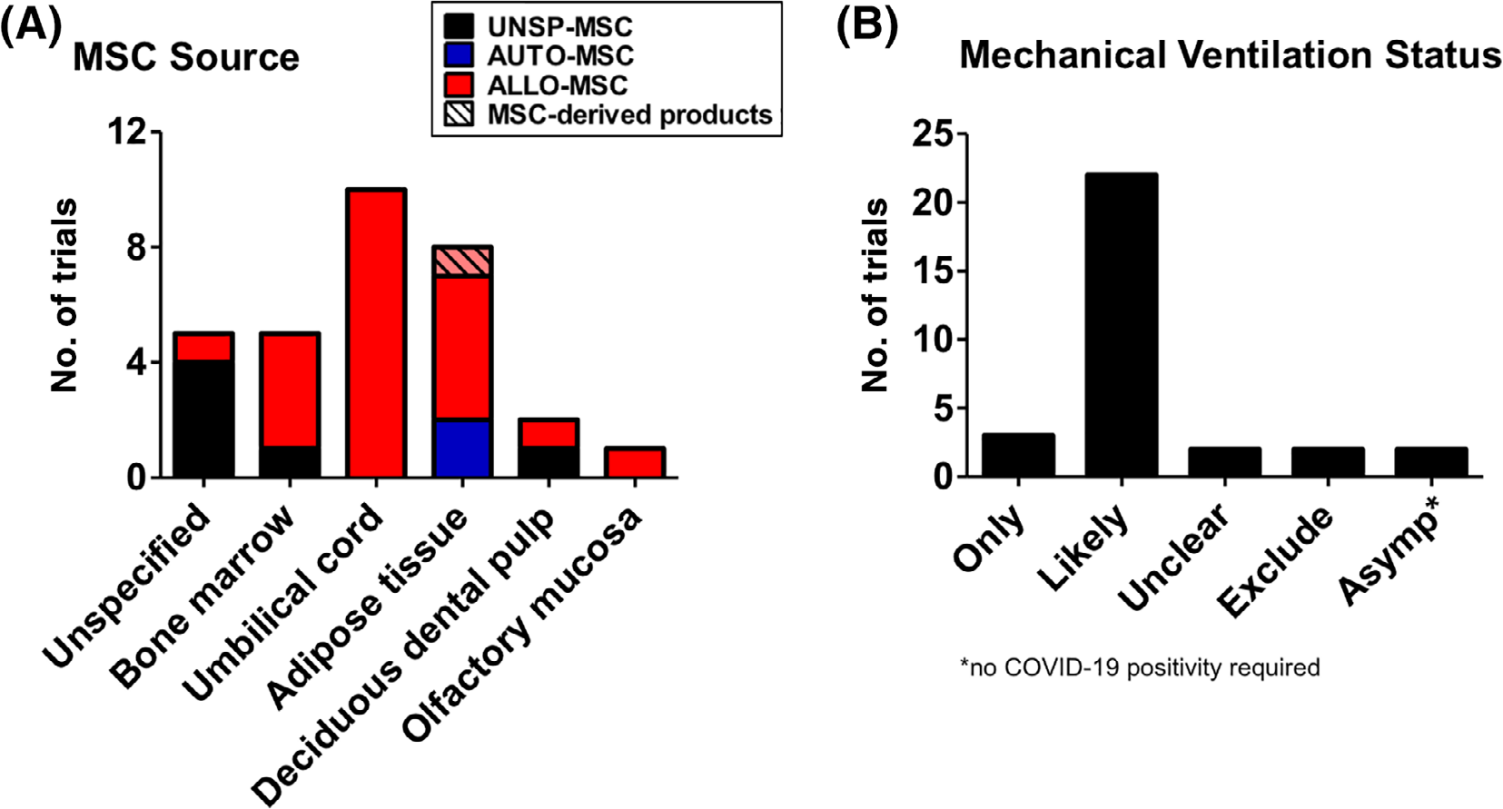
Sources of MSCs used and mechanical ventilation status of patients in COVID‐19 clinical trials. A, Number of trials using different sources of human MSCs is shown, and whether sources are autologous (AUTO‐MSC), allogeneic (ALLO‐MSC), unspecified (UNSP‐MSC), and/or noncell exosomes/conditioned medium (MSC‐derived products). B, Number of trials with regards to mechanical ventilation status for patient inclusion in COVID‐19 trials. Three trials have mechanical ventilation as an inclusion criteria (ONLY); 22 trials have inclusion criteria which would only capture hospitalized/severely ill patients (LIKELY), that is, including patients in the ICU and those who requiring exogenous oxygen and/or intubation; two trials only have COVID‐19 positivity as inclusion criteria so it is unclear whether very ill, mechanically ventilated patients would be included (UNCLEAR). Two trials exclude intubated patients (EXCLUDE), and two trials include asymptomatic individuals (ASYMP). All data accessed on May 2020 from the NIH Clinical Trial website (https://ClinicalTrials.gov/). ICU, intensive care unit; MSC, mesenchymal stem cell

There are 31 MSC trials specifically targeting COVID‐19 registered on the NIH Clinical Trial website as of 17 May 2020 (Table 2). These trials are located globally, with 11 in China, 8 in the United States, 9 in Western Europe (4 in Spain, and 1 each in the United Kingdom, France, Germany, Denmark, and Belarus), 2 in the Middle East, and 1 in Brazil (Table S2 with brief details for these trials). Surprisingly, 74% of the trials use allogeneic MSCs (23 trials) with only two trials using autologous sources (AdMSCs in both trials); the remaining six trials were unspecified (Figure 5A). The overwhelming use of allogeneic cells is likely due to the rapid patient deterioration which would probably not allow for autologous harvesting and the 1 to 3 weeks needed for MSCs to emerge in culture. UCMSCs are the predominant source used (10 trials), with AdMSCs being the next most used source (seven trials) and BMMSCs used in five trials. Dental pulp MSCs are used in two trials, and one trial use olfactory mucosa MSCs; five trials did not specify type of MSCs. Only one trial using exosomes derived from allogeneic AdMSCs will use inhalation as the route of delivery, otherwise in all other trials MSCs will be infused intravenously; one trial did not specify this detail. Unlike for other disease entities, the trials for COVID‐19 are more equally distributed, with eight trials in phase 1, nine trials each in phase 1+2 and phase 2. There are trials in later phases as well, with two being phase 2+3, and one in phase 3; two trials did not specify phase. Two of the phase 2 trials are unusual, however, since individuals who are asymptomatic are targeted, without requiring COVID‐19‐positivity as an inclusion criteria. Further examination revealed noteworthy details as stated in the trials: in these two trials which are sponsored by the same commercial entity, AdMSCs will be used as prevention against COVID‐19 infection, with allogeneic AdMSCs used for high‐risk populations such as health workers and other essential workers, and autologous AdMSCs for the original donors more than 65 years of age. Given the strong immunomodulatory effect of MSCs and the clinical report of coincident bacterial pneumonia after MSC infusion,^86^ use of MSC treatment as a preventive measure against a novel virus should be rigorously evaluated, at the very least. Except for these two trials, all other trials require COVID‐19 positivity for inclusion, with the vast majority of the trials only accepting severely ill patients (Figure 5B): three trials have mechanical ventilation as an inclusion criteria, and 22 trials have inclusion criteria which would only capture hospitalized/severely ill patients, that is, including patients in the intensive care unit (ICU) and those who requiring exogenous oxygen and/or intubation. Two trials only require COVID‐19 positivity, and two other trials oddly excluded intubated patients. An encouraging point is that a majority of the trials will be randomized (21 trials). In addition to these MSCT trials that specifically target COVID‐19, there are four trials of MSCT for ARDS and/or severe viral pneumonia which were just posted in the past few months (NCT3818854, NCT04282928, NCT04289194, and NCT04347967—see Table S152 for trial details). While it is unclear whether the rapid increase in MSCT trials for COVID‐19 is a factor in accelerating the registration of these trials, there is strong interest in using MSCT for severe pulmonary inflammation and complications given the continued lack of curative treatments for ARDS from any cause.

**TABLE 2:** Cell source and trial phase of MSC clinical trials for COVID‐19

MSC source	Total %	Total no.	No. of clinical trial phases					
			?	1	1&2	2	2&3	3
Unspecified	16.1	5	0	2	0	2	1	0
Bone marrow	16.1	5	0	1	1	1	1	1
Umbilical cord	32.3	10	2	2	4	2	0	0
Adipose tissue	22.6	7	0	1	2	4	0	0
Deciduous dental pulp	6.5	2	0	1	1	0	0	0
Olfactory mucosa	3.2	1	0	0	1	0	0	0
MSC‐derived products^a^	3.2	1	0	1	0	0	0	0
Total no. of clinical trial phases		31	2	8	9	9	2	1
Total % of clinical trial phases			6.5	25.8	29.0	29.0	6.5	3.2

^a^ Exosomes collected from conditioned medium.

Abbreviation: MSC, mesenchymal stem cell.

As testament to the rapid development in all COVID‐19‐related matters, just 2 months ago in February 2020, a report of clinical MSCT for COVID‐19 was published.^93^ Held in China, this small clinical trial evaluated a single infusion of BMMSCs in seven patients aged 45 to 65 with mild to critically severe COVID‐19 who did not improve with standard treatments; the BMMSCs were obtained from a commercial entity so are likely to be allogeneic, but this was not specified in the report. No infused patients were mechanically ventilated, although the one critically severe case patient was in the ICU; three non‐ICU patients (one aged 46, and two aged 74‐75) with severe disease were used as controls. All seven patients receiving MSC infusion recovered, but for the three control patients, there was one death. Immunologically, patients who received MSCs compared to placebo controls had significantly lower levels of TNF‐α and higher levels of IL‐10, but strangely, only three patients in the MSC group were included in these analyses. Patients who received MSCs were measured before and after treatment with regards to Tregs and immunomodulatory dendritic cells, with results demonstrating that only severely and critically ill patients had increases of these immunomodulatory immune cells. No analysis of age on outcome was performed. While the small sample size precludes more in‐depth analyses, these results appear to trend similarly to findings from the preclinical influenza animal studies, in which MSCT efficacy is more apparent with more severe disease/pathology.

## 7 PERSPECTIVE ON MSCT FOR COVID‐19

Given the consistent beneficial outcomes of MSCT in the many preclinical in vivo studies of bacteria‐induced ARDS, tempered by the smaller data set of MSC‐virus interactions which include two clinical reports, any consideration of MSCT for COVID‐19 should be steered toward very severe cases where ARDS and an exuberant immune response is seen and not during the early period of viral infection or in mild cases. Like the previous two other novel corona viruses which have caused severe disease, COVID‐19‐ARDS is accompanied by cytokine storm and severe inflammation.^94^ The preclinical data on MSCT decreasing TNF‐α and IL‐6 levels, two pro‐inflammatory cytokines highly expressed during cytokine storm,^95^ have been quite consistent, and clinical MSCT data from the other immune/inflammatory diseases—particularly GVHD which have had long follow‐up periods—as well as the two recently published viral pneumonia trials, support the relative safety of MSCT even if efficacy may be more difficult to interpret. In light of the continued increase of COVID‐19 cases and deaths, there has been an avalanche of false information exploiting patients and the public during these uncertain times. The leading stem cell and cell therapy academic societies have all issued statements of caution against unproven stem cell treatments, emphasizing the importance of testing new possible therapies in clinical trials first.^96^, ^97^ The prospect of MSCT for COVID‐19, therefore, must be tempered with strict evaluation of patient inclusion/exclusion criteria as well as stringent ethical consideration to foremost protect patient safety.

## CONFLICT OF INTEREST

The authors declared no potential conflicts of interest.

## AUTHOR CONTRIBUTIONS

B.L.Y.: conception, manuscript writing, final approval, funding; M.‐L.Y.: conception, manuscript writing, final approval; L.‐T.W., K.‐J.L., H.‐K.S.: manuscript writing.

## Supporting information


**Supplementary Table S1** List of current MSC clinical trials for immune/inflammatory lung diseases.

**Supplemental Table S2** Brief description of current COVID‐19 MSC trials (detailed information on each trial can be found in the Supplemental Table)

## Data Availability

Data sharing is not applicable to this article as no new data were created or analyzed in this study.
