# Supplementary material for: Current status of mesenchymal stem cell therapy for immune/inflammatory lung disorders: Gleaning insights for possible use in COVID‐19
Source: Stem Cells Transl Med. 2020 Jun 11;9(10):1163–73. doi: 10.1002/sctm.20-0186 (PMC7300965; doi:10.1002/sctm.20-0186)
Supplement: Supplementary file 2 — Supplemental Table S2 Brief description of current COVID‐19 MSC trials (detailed information on each trial can be found in the Supplemental Table) [file SCT3-9-1163-s002.docx]

**Table 3: Brief description of current COVID-19 MSC trials (detailed information on each trial can be found in the Supplemental Table)**

| ***NCT Number*** | ***Location*** | ***Inclusion Criteria*** | ***Source MSCs*** | ***Auto/Allo?*** | ***Delivery*** | ***Phase*** | ***Randomized?*** |
| --- | --- | --- | --- | --- | --- | --- | --- |
| NCT03042143 | Europe (UK) | Intubated only | UCMSCs | Allogeneic | IV | 1 + 2 | Yes |
| NCT04252118 | China | Unclear: PCR(+) & pneum on x-ray/CT | Unspec | Unspec | IV | 1 | No |
| NCT04269525 | China | Likely: include severe pneum & in ICU | UCMSCs | Allogeneic | IV | 2 | No |
| NCT04273646 | China | Likely: include severe pneum | UCMSCs | Allogeneic | IV | Unspec | Yes |
| NCT04276987 | China | Likely: include severe to critical pneum | AdMSC-exos | Allogeneic | Inhaled | 1 | No |
| NCT04288102 | China | Exclude interventional ventilation | Unspec | Unspec | IV | 2 | Yes |
| NCT04293692 | China | Likely: include mild to severe pneum | UCMSCs | Allogeneic | IV | Unspec | Yes |
| NCT04302519 | China | Likely: include severe pneum | DPMSCs | Unspec | IV | Early 1 | No |
| NCT04313322 | Middle East | Unclear: PCR(+) only | UCMSCs | Allogeneic | IV | 1 | No |
| NCT04315987 | Brazil | Likely: <50% lung function | Unspec | Unspec | IV | 1 | No |
| NCT04333368 | Europe (France) | Likely: ARDS | UCMSCs | Allogeneic | IV | 1 + 2 | Yes |
| NCT04336254 | China | Likely: include severe pneum | DPMSCs | Allogeneic | IV | 1 + 2 | Yes |
| NCT04339660 | China | Likely: include mild to severe pneum | UCMSCs | Allogeneic | IV | 1 + 2 | Yes |
| NCT04341610 | Europe (Denmark) | Likely: include severe pneum | AdMSCs | Allogeneic | Unspec | 1 + 2 | Yes |
| NCT04345601 | USA | Likely: Mild to severe ARDS | BMMSCs | Allogeneic | IV | Early 1 | No |
| NCT04346368 | China | Likely: include severe pneum | BMMSCs | Unspec | IV | 1 + 2 | Yes |
| NCT04348435 | USA | Asymptomatic (PCR status not mentioned) | AdMSCs | Allogeneic | IV | 2 (preven) | Yes |
| NCT04348461 | Europe (Spain) | Intubated only | AdMSCs | Allogeneic | IV | 2 | Yes |
| NCT04349631 | USA | Asymptomatic (PCR status not mentioned) | AdMSCs | Autologous | IV | 2 (preven) | No |
| NCT04352803 | USA | Likely: hospitalized & exclude mild disease | AdMSCs | Autologous | IV | 1 | No |
| NCT04355728 | USA | Intubated only | UCMSCs | Allogeneic | IV | 1 + 2 | Yes |
| NCT04361942 | Europe (Spain) | Likely: include severe pneum & in ICU | Unspec | Allogeneic | IV | 2 | Yes |
| NCT04362189 | USA | Likely: hospitalized | AdMSCs | Allogeneic | IV | 2 | Yes |
| NCT04366063 | Middle East | Likely: ARDS | Unspec | Unspec | IV | 2 + 3 | Yes |
| NCT04366271 | Europe (Spain) | Exclude interventional ventilation | UCMSCs | Allogeneic | IV | 2 | Yes |
| NCT04366323 | Europe (Spain) | Likely: include severe to critical pneum | AdMSCs | Allogeneic | IV | 1 + 2 | Yes |
| NCT04367077 | USA | Likely: include moderate to severe pneum | BM (MAPCs) | Allogeneic | IV | 2 + 3 | Yes |
| NCT04371393 | USA | Likely: ARDS | BMMSCs | Allogeneic | IV | 3 | Yes |
| NCT04371601 | China | Likely: include severe to critical pneum | UCMSCs | Allogeneic | IV | Early 1 | Yes |
| NCT04377334 | Europe (Germany) | Likely: require exogenous oxygen | BMMSCs | Allogeneic | IV | 2 | Yes |
| NCT04382547 | Europe (Belarus) | Likely: respiratory failure | OMMSCs | Allogeneic | IV | 1 + 2 | No |

New abbreviations: pneum, pneumonia; DPMSCs, dental pulp MSCs; OMMSCs, olfactory mucosa MSCs; Unspec, unspecified; MAPCs, multipotent adult progenitor cells; IV, intravenous; preven, preventive.
